# Supplementary material for: Adaptive tuning of mutation rates allows fast response to lethal stress in Escherichia coli
Source: eLife. 2017 May 2;6:e22939. doi: 10.7554/eLife.22939 (PMC5429094; doi:10.7554/eLife.22939)
Supplement: Supplementary file 1. — DOI: http://dx.doi.org/10.7554/eLife.22939.024 [file elife-22939-supp1.docx]

**Supplementary file 1: mutator genes and used primers**

**Supplementary file 1A**: Selected genes conferring a mutator phenotype and their corresponding Keio collection number

| **Gene** | **Function** | **Keio Collection Number^84^** |
| --- | --- | --- |
| *xthA* | Abasic lesion (AP-site) endonuclease | JW1738 |
| *mutM* | DNA glycosylase, base excision repair | JW3610 |
| *mutY* | Adenine glycosylase, GA mismatch | JW2928 |
| *mutT* | 8-oxo-dGTP diphosphatase | JW0097 |
| *mutS^*^* | Methyl directed mismatch repair | JW2703 |
| *mutL* | Methyl directed mismatch repair | JW4128 |
| *mutH* | Methyl directed mismatch repair | JW2799 |
| *uvrD* | Helicase involved in MMR and BER | JW3786 |
| *dnaQ* | Proofreading subunit of DNA polymerase III | JW0205 |

*^*^∆*mutS *was found to be inaccurate in the Keio collection and was therefore separately constructed using the Red-mediated homologous recombination technique (see Methods).*

**Supplementary file 1B**: Overview of used primer sequences in the construction of the mutator mutants

| **Primer sequence** | **Function** |
| --- | --- |
|  |  |
| GAAATTACTGCGCCATTCTGAC | Forward primer to check *xthA* deletion |
| GTGGTCAGACAACTAAAAACGC | Reverse primer to check *xthA* deletion |
| AATCGCCACTTAATGCCGGA | Forward primer to check *mutM* deletion |
| AAACGTACTAAGCCGGAAAAACT | Reverse primer to check *mutM* deletion |
| GGCCAAAAAGCGCGGGG | Forward primer to check *mutY* deletion |
| GGGGTACAGCTGAAAATCCTGAC | Reverse primer to check *mutY* deletion |
| **AATAAAAACCATCACACCCCATTTAATATCAGGGAACCGGACATAACCCC**GTGTAGGCTGGAGCTGCTTC | Forward primer for ∆*mutS* construction*^*^* |
| **GTCAGTTGTCGTTAATATTCCCGATAGCAAAAGACTATCGGGAATTGTTA**CATATGAATATCCTCCTTA | Reverse primer for ∆*mutS* construction*^*^* |
| GCGCCTTATGTGATTACAACG | Forward primer to check *mutS* deletion |
| GATAGCGTGTAGATGGCATGG | Reverse primer to check *mutS* deletion |
| AGTACGCTGAGCAAAGTTC | Forward primer to check *mutT* deletion |
| ACGAATCCCCAGCAGTG | Reverse primer to check *mutT* deletion |
| AACCGGCATTTGTGTTGTATC | Forward primer to check *mutL* deletion |
| CTTGCTCTGCCGCCTGT | Reverse primer to check *mutL* deletion |
| GTACTTTTTGGCTGCGGAAAAAC | Forward primer to check *uvrD* deletion |
| GTCGAGCGGAAAGGTTAAAACG | Reverse primer to check *uvrD* deletion |
| TCAAGGTATCATGACATGTCCC | Forward primer to check *mutH* deletion |
| GGAAAGCGGCAGGTCAAA | Reverse primer to check *mutH* deletion |
| CGCTAAAGGTTTTCTCGCGT | Forward primer to check *dnaQ* deletion |
| CGGATTGCCTCGACCTTC | Reverse primer to check *dnaQ* deletion |
|  |  |

*^*^ Bold part of the primer shows the part homologous to the genomic region up- and downstream of the* mutS *gene; Underlined part shows the priming part binding on the pKD4 plasmid to amplify the kanamycin resistance cassette flanked by FRT sites (see methods).*
